# Understanding Hospital-Level Patterns of Nonoperative Management for Low-risk Thyroid and Kidney Cancer

**DOI:** 10.1001/jamanetworkopen.2022.42210

**Published:** 2022-11-15

**Authors:** Mara Koelker, Marieke Krimphove, Khalid Alkhatib, Junaid Nabi, Lindsay E. Kuo, Stuart R. Lipsitz, Toni K. Choueiri, Steven Lee Chang, Gerard M. Doherty, Adam S. Kibel, Quoc-Dien Trinh, Alexander P. Cole

**Affiliations:** 1 Center of Surgery and Public Health, Division of Urological Surgery, Brigham and Women’s Hospital, Boston, Massachusetts; 2 Department of Urology, University Medical Center Hamburg-Eppendorf, Hamburg, Germany; 3 Department of Urology, University Hospital Frankfurt, Goethe University Frankfurt am Main, Frankfurt, Germany; 4 Department of Surgery, Temple University Lewis Katz School of Medicine, Philadelphia, Pennsylvania; 5 Lank Center for Genitourinary Oncology, Dana Farber Cancer Institute, Harvard Medical School, Boston, Massachusetts; 6 Department of Surgery, Brigham and Women's Hospital, Harvard Medical School, Boston, Massachusetts; 7 Division of Urological Surgery, Brigham and Women's Hospital, Harvard Medical School, Boston, Massachusetts

## Abstract

**Importance:**

There is a growing trend toward conservative management for certain low-risk cancers. Hospital and health-system factors may play a role in determining how these patients are managed.

**Objective:**

To explore the contribution of hospitals on patients’ odds of nonoperative management for low-risk cancer.

**Design, Setting, and Participants:**

In this cross-sectional study, individuals with low-risk papillary thyroid cancer and solitary kidney masses were identified, and those receiving nonoperative management vs surgery were compared. Patients with low-risk thyroid cancer and kidney cancer from 2015 to 2017 eligible for nonoperative management according to National Comprehensive Cancer Network guidelines within the National Cancer Database were included. Data were analyzed from October 2021 to March 2022.

**Main Outcomes and Measures:**

For each facility, the proportion of these patients who received operative and nonoperative management was calculated. A mixed-effects logistic regression model with a hospital-level random effects term was used to calculate factors associated with nonoperative management. Between-hospital variability was assessed using ranked caterpillar plots.

**Results:**

There were 19 570 individuals with low-risk thyroid cancer (15 344 women [78.4%]; mean [SD] age, 51.74 [95% CI, 51.39-52.08] years) and 41 403 with kidney cancer (25 253 men [61.0%]; mean [SD] age, 61.93 [95% CI, 61.70-62.17] years). In the group with low-risk thyroid cancer, 2.1% (419 patients) received nonoperative management, and in the group with kidney cancer, 9.5% (3928 patients) received nonoperative management. This varied between hospitals from 1.1% (95% CI, 1.0%-1.1%) in the bottom decile to 10.3% (95% CI, 8.0%-12.4%) in the top decile for low-risk thyroid cancer, and from 4.3% (95% CI, 4.1%-4.4%) in the bottom decile to 24.6% (95% CI, 22.7%-26.5%) in the top decile for small kidney masses. For both cancers, age was associated with increased odds of nonoperative treatment. The hospital-level odds of nonoperative management of thyroid and kidney cancer using unadjusted probabilities (observed proportions) were minimally correlated (Spearman ρ = .33; *P* < .001).

**Conclusions and Relevance:**

The findings of this study suggest that although health systems factors may be associated with the tendency to pursue nonoperative management, hospital-level factors may differ when comparing unrelated cancers.

## Key Points

**Question:**

Are hospital and health system factors associated with nonoperative management for patients with low-risk thyroid cancer and small kidney masses?

**Findings:**

In this cross-sectional study of 19 570 individuals with thyroid cancer and 41 403 individuals with kidney cancer, nonoperative management was used in a minority of cases. The hospital-level odds of nonoperative management using unadjusted probabilities were minimally correlated between the 2 cancers.

**Meaning:**

These findings suggest that health systems factors may be associated with the tendency to pursue nonoperative management, but the specific factors may differ between these 2 unrelated cancers.

## Teaser

This cross-sectional study evaluates hospital-level patterns of nonoperative management for low-risk thyroid and kidney cancer.

## Introduction

Although high-intensity local therapy such as radical surgery and radiation remain the standard of care for most localized malignant neoplasms, there is a growing set of cancers for which less intensive treatment approaches are increasingly used. These management strategies include observation (active surveillance), focal therapy, and less intensive surgical approaches (eg, organ-sparing surgical procedures), as well as some forms of observation with the potential for deferred intervention. This paradigm shift likely represents the confluence of 2 important trends. First, there is a growing body of epidemiological evidence that large numbers of men and women with small and potentially slow-growing cancers may be overtreated, leading to adverse effects of treatment without changing the risk of death.^1^ Second, improvements in imaging and understanding of tumor biology have helped to identify scenarios in which less intensive treatments can be safely used.^2^ Examples for observation include small kidney masses (vs nephrectomy or partial nephrectomy)^3^; papillary thyroid cancer, which since 2015 can be managed with observation or partial thyroidectomy (vs total thyroidectomy with or without lymphadenectomy)^4,5^; and ductal breast carcinoma in situ (vs mastectomy or lumpectomy).^6^

Although each cancer has its own set of oncologic factors that may guide nonoperative management vs surgical therapy, there also may be hospital and health systems factors that are associated with patients’ chances of receiving more or less aggressive treatment. These factors include geography, local practice patterns, payment models, income, and even distance from the treating hospital.^7^ Although there is well-described variation in the use of nonoperative management for some cancers,^8,9^ less is known about whether these same factors apply to different cancers. For example, health systems that pay surgeons flat salaries may offer more nonoperative management than those where surgeons are paid according to case volume.

To better understand the health systems factors that may be associated with the probability of nonoperative management for low-risk cancer, and to assess whether these factors apply to unrelated cancers, we designed a retrospective, registry-based study to correlate hospital-level patterns of nonoperative management in kidney and thyroid cancer. We hypothesized that there is significant variability in hospitals’ rates of nonsurgical treatment for thyroid and kidney cancer. Furthermore, we hypothesized that hospitals more likely to use nonoperative management for thyroid cancer would also be more likely to pursue nonoperative management for kidney cancer and vice versa.

## Methods

### Data Source

For the reporting of the study we followed the Strengthening the Reporting of Observational Studies in Epidemiology (STROBE) reporting guideline for cross-sectional studies.^10^ The study was approved by the Brigham and Women’s Hospital institutional review board. The study utilized anonymous cancer registry data so informed consent was not required. Data were obtained from the National Cancer Database (NCDB), a comprehensive cancer registry established by the Commission on Cancer of the American Cancer Society. This database includes patients seen at 1 of 1500 participating Commission on Cancer–accredited hospitals for any portion of diagnosis or treatment. The database captures a large portion of incident cancer in the US, including 92.1% of thyroid cancer cases and 86.7% of all kidney cancer.^11^ Trained data abstractors use a standardized method to collect sociodemographic and clinical data (eg, tumor site, stage, grade, and treatment type) as reported by participating institutions. The database is considered to have good capture of clinical, pathologic, and treatment data, and the facility identification data within the database are consistent across cancers, allowing for between-hospital comparisons.^12^

### Study Population

Patients with a new diagnosis of papillary thyroid or kidney cancer from 2015 to 2017 within the National Cancer Database were selected. For each patient, age at diagnosis, sex, race and ethnicity, insurance status, family income, and tumor stage were assessed. These dates were chosen because 2015 was the year in which a nonoperative option for localized thyroid cancer was added to the National Comprehensive Cancer Network (NCCN) guidelines.^13^

For each of the 2 malignant neoplasms, stage and grade criteria were used to generate an analytic cohort of patients who meet the criteria for nonoperative management. Specifically, for low-risk papillary thyroid cancer less than 1cm, management options are lobectomy alone possibly followed by completion thyroidectomy (high-intensity) with or without lymphadenectomy or active surveillance (nonoperative management). Similarly, small (T1a) or lower kidney masses could be managed with surveillance or focal therapy or radical or partial nephrectomy (surgery). Only hospitals with at least 25 men and women eligible for nonoperative management in both cancers were included. Detailed summaries of the definitions for surgical and nonsurgical treatment are available in eTable 1 in the Supplement.

### Covariates

The following sociodemographic covariates were included: age at diagnosis, race as provided by the NCDB (White, Black, Asian American, Native Hawaiian, and Pacific Islander, and other, which includes other and unknown as defined within NCDB), primary insurance type (private, Medicaid, Medicare, and other, including other government payer [including TRICARE, Military, VA, and Indian/Public Health Service]) and distance from patients’ home zip (postal) code to the treating hospital. Race was assessed given well documented associations of race with worse survival and lower odds of definitive treatment for some cancers.^14,15^ Clinical comorbidities were estimated by using the Charlson-Deyo modification of Charlson Comorbidity Index score (CCI; 0, 1, >2; score predicts 10-year survival in patients with multiple comorbidities, and comorbidties can be ranked 1-6).^16^

### Outcome Variable

For our dichotomous main outcome variable “nonoperative management,” we used the variable rx_summ_treatment_status. This variable summarizes whether the patient received any treatment or was under active surveillance.^17^ Men and women whose primary treatment was coded as “no surgery” and “active surveillance” were categorized in the nonoperative group, and those whose primary treatment was coded using other than these 2 variables were categorized as undergoing “operative management.” We chose to include all men and women undergoing nonoperative management as a primary treatment strategy in addition to those specifically treated with active surveillance as we felt this provided the most comprehensive picture of overall nonoperative management for these cancers.

### Statistical Analysis

For each of the 2 malignant neoplasms, we used descriptive statistics to compare patients receiving nonoperative management vs those who received primary treatment with surgery. Baseline characteristics between the groups were compared with the χ^2^ test (categorical variables) and standardized differences (continuous variables).

For the 2 cancers, we fit a multivariable mixed-effects logistic regression to identify the association between nonsurgical management and patient characteristics. Additionally, a sensitivity analysis using generalized estimating equations was conducted. First, an unadjusted mixed-effects logistic regression with only hospital random effects and fixed cancer type was conducted. Second, a model with hospital-level intercept (random effects) was used to account for the contribution of each individual hospital on their patients’ probability of nonsurgical therapy. Given less than 10% of missingness in variables, missing values for covariates were ignored as these have been shown to have low probability of skewing results.^18^ Missing outcome variables (>5%) were assumed to be nonignorable, and a maximum likelihood technique for our multilevel model was used to address this.^19^

For each hospital, a risk-adjusted probability of nonoperative management with 95% CIs was calculated. After holding patient-level covariates (fixed effects) at their frequency in the overall population, we estimated each hospital’s rate of nonoperative management according to the hospital-level random intercepts. To calculate the estimated probabilities for a hospital, on the logit scale for each covariate we used the population mean value plus the random hospital effect. We then transformed from the logit scale to the probability scale. Thus, each hospital has the same set of covariate values, the population mean. The only difference is the random effect for each hospital. Hospitals were ranked from least likely to most likely to perform nonoperative treatment.^20,21^ The mean estimated probability of observation in the top and bottom deciles with 95% CIs were compared.

Finally, we assessed whether unadjusted hospitals’ probability (observed proportions) of nonoperative management was correlated between the 2 cancers. Scatterplots were generated where each hospitals’ estimated probability of observation in thyroid cancer was plotted against that hospitals’ probability of observation in kidney cancer. Spearman rank coefficient was calculated to assess the correlation between hospitals’ probability of observation in kidney and thyroid cancer.

Two-sided statistical significance was defined as *P* < .05. All statistical analyses were performed using Stata statistical software version 14.0 (StataCorp). Data were analyzed from October 2021 to March 2022.

## Results

After applying the inclusion criteria outlined previously, of 112 735 patients, there were 19 570 (15 344 [78.4%] women) with low-risk papillary thyroid cancer and papillary microcarcinoma that could be eligible for nonoperative management according to NCCN guidelines with an mean age of 51.74 years (95% CI, 51.39-52.08 years) in the thyroid cohort. Of these, 419 (2.1%) received nonoperative management and 19 151 (97.9%) received surgery. Of a total of 149 446 patients, there were 41 403 (25 253 [61.0%] men) with small kidney masses who were eligible for surveillance according to NCCN guidelines with a mean age of 61.93 (95% CI, 61.70-62.17) years. Of these, 3928 (9.5%) received nonoperative management and 37 475 (90.5%) received surgery treatment. The clinical and demographic characteristics of the kidney and thyroid cancer cohorts are summarized in Table 1.

**Table 1.** Baseline Characteristics of Men and Women Eligible for Nonsurgical Management Strategies for Low-risk Thyroid and Kidney Cancer in the National Cancer Database From 2015 to 2017

Characteristic	Papillary thyroid cancer				Small kidney masses			
	Total (N = 19 570)	Surgical treatment (n = 19 151 [97.9%])	Nonoperative management (n = 419 [2.1%])	*P* value^a^	Total (N = 41 403)	Surgical treatment (n = 37 475 [90.5%])	Nonoperative management (n = 3928 [9.5%])	*P* value^a^
Sex								
Male	4226 (21.6)	4108 (21.5)	118 (28.2)	.03	25 253 (61.0)	22 862 (61.0)	2391 (60.9)	.89
Female	15 344 (78.4)	15 043 (78.6)	301 (71.8)		16 150 (39.0)	14 613 (39.0)	1537 (39.1)	
Age group, y								
≤49	8339 (42.6)	8175 (42.7)	164 (39.1)	<.001	6859 (16.6)	6607 (17.6)	252 (6.4)	<.001
50-59	5163 (26.4)	5071 (26.5)	92 (22.0)		9525 (23.0)	9019 (24.1)	506 (12.9)	
60-69	3953 (20.2)	3867 (20.2)	86 (20.5)		13 172 (31.8)	12 142 (32.4)	1030 (26.2)	
70-79	1744 (8.9)	1701 (8.9)	43 (10.3)		8927 (21.6)	7744 (20.7)	1183 (30.1)	
≥80	371 (1.9)	337 (1.8)	34 (8.1)		2920 (7.1)	1963 (5.2)	957 (24.4)	
Race								
Asian American, Native Hawaiian, and Pacific Islander	1296 (6.6)	1264 (6.6)	32 (7.6)	.13	1086 (2.6)	1004 (2.7)	82 (2.1)	<.001
Black	1651 (8.4)	1624 (8.5)	27 (6.4)		5910 (14.3)	5189 (13.9)	721 (18.4)	
White	15 834 (80.9)	15 500 (80.9)	334 (79.7)		33 521 (81.0)	30 468 (81.3)	3053 (77.7)	
Other^b^	789 (4.0)	763 (4.0)	26 (6.2)		886 (2.1)	814 (2.2)	72 (1.8)	
Charlson Comorbidity Index								
0	16 156 (82.6)	15 788 (82.4)	368 (87.8)	.01	27 606 (66.7)	24 996 (66.7)	2610 (66.5)	.01
1	2605 (13.3)	2567 (13.4)	38 (9.1)		7899 (19.1)	7221 (19.3)	678 (17.3)	
≥2	809 (4.1)	796 (4.2)	13 (3.1)		5898 (14.3)	5258 (14.0)	640 (16.3)	
Insurance								
Private	12 842 (65.6)	12 584 (65.7)	258 (61.6)	.08	17 130 (41.4)	16 238 (43.3)	892 (22.7)	<.001
Medicare	4084 (20.9)	3977 (20.8)	107 (25.5)		19 277 (46.6)	16 677 (44.5)	2600 (66.2)	
Medicaid	1762 (9.0)	3977 (9.0)	30 (7.2)		2975 (7.2)	2737 (7.3)	238 (6.1)	
Other	882 (4.5)	858 (4.5)	24 (5.7)		2021 (4.9)	1823 (4.9)	198 (5.0)	
Annual household income, $								
>63 000	7390 (37.8)	7195 (37.6)	195 (46.5)	.07	11 393 (27.5)	10 367 (27.7)	1026 (26.1)	.06
48 000-62 999	4187 (21.4)	4106 (21.4)	81 (19.3)		9395 (22.7)	8515 (22.7)	880 (22.4)	
38 000-47 999	3206 (16.4)	3139 (16.4)	67 (16.0)		8136 (19.7)	7378 (19.7)	758 (19.3)	
<38 000	2202 (11.3)	2163 (11.3)	39 (9.3)		6418 (15.5)	5709 (15.2)	709 (18.1)	
Unknown	2585 (13.2)	2548 (13.3)	37 (8.8)		6061 (14.6)	5506 (14.7)	555 (14.1)	
Distance to hospital, miles								
0-12.4	8383 (42.8)	8152 (42.6)	231 (55.1)	.00	16 356 (39.5)	14 535 (38.8)	1821 (46.4)	<.001
12.5-49.9	6504 (33.2)	6405 (33.4)	99 (23.6)		13 034 (31.5)	11 945 (31.9)	1089 (27.7)	
≥50	2117 (10.8)	2065 (10.8)	52 (12.4)		5985 (14.5)	5518 (14.7)	467 (11.9)	
Unknown	2566 (13.1)	2529 (13.2)	37 (8.8)		6028 (14.6)	5477 (14.6)	551 (14.0)	

^a^ Wald test with hospital-level clustering; Pearson χ^2^ test was used to test significance.

^b^ Other includes other and unknown as defined within the National Cancer Database.

Independent patient and hospital-level factors associated with nonoperative management are listed in Table 2. For thyroid cancer, age older than 70 years (odds ratio [OR], 1.69; 95% CI, 1.07-2.67) was independently associated with higher odds of nonoperative management. Female sex (OR, 0.77; 95% CI, 0.61-0.98) and a low CCI score of 1 (OR, 0.59; 95% CI, 0.41-0.85) were associated with lower odds of nonoperative management. The sensitivity analysis is displayed in eTable 2 in the Supplement.

**Table 2.** Patient-Level Factors Associated With Nonsurgical Management in Eligible Patients With Low-risk Papillary Thyroid Cancer and Small Kidney Masses Eligible for Surveillance by NCCN Guidelines

Characteristic	Papillary thyroid cancer		Solitary kidney masses	
	OR (95% CI)	*P* value^a^	OR (95% CI)	*P* value^a^
Sex				
Male	1 [Reference]	.04	1 [Reference]	.39
Female	0.77 (0.61-0.98)		0.96 (0.88-1.05)	
Age group, y				
≤49	1 [Reference]	NA	1 [Reference]	NA
50-59	1.04 (0.79-1.38)	.77	1.46 (1.22-1.76)	<.001
60-69	1.35 (0.98-1.85)	.06	2.20 (1.84-2.62)	<.001
70-79	1.69 (1.07-2.67)	.03	4.10 (3.40-4.94)	<.001
80-89	7.11 (4.15-12.19)	<.001	14.19 (11.56-17.41)	<.001
Race				
White	1 [Reference]	NA	1 [Reference]	NA
Asian American, Native Hawaiian, and Pacific Islander	1.15 (0.76-1.73)	.51	0.76 (0.56-1.04)	.09
Black	0.95 (0.61-1.47)	.82	1.49 (1.32-1.69)	<.001
Other^b^	1.31 (0.83-2.07)	.25	0.96 (0.71-1.30)	.80
Charlson Comorbidity Index				
0	1 [Reference]	NA	1 [Reference]	NA
1	0.59 (0.41-0.85)	.01	0.76 (0.68-0.86)	<.001
≥2	0.72 (0.39-1.33)	.29	0.83 (0.73-0.93)	.00
Insurance				
Private	1 [Reference]	NA	1 [Reference]	NA
Medicare	1.02 (0.73-1.46)	.88	1.33 (1.18-1.50)	<.001
Medicaid	1.25 (0.81-1.94)	.32	1.57 (1.30-1.90)	<.001
Other	1.55 (0.96-2.51)	.07	1.32 (1.06-1.65)	.01
Annual household income, $				
>63 000	1 [Reference]	NA	1 [Reference]	NA
48 000-62 999	0.82 (0.61-1.12)	.22	1.05 (0.93-1.20)	.42
38 000-47 999	1.03 (0.75-1.43)	.85	1.10 (0.95-1.26)	.20
<38 000	0.76 (0.51-1.15)	.20	1.24 (1.07-1.44)	.01
Unknown	0.33 (0.00-50.14)	.66	1.55 (0.30-8.09)	.61
Distance to hospital, miles				
0-12.4	1 [Reference]	NA	1 [Reference]	NA
12.5-49.9	0.42 (0.32-0.55)	<.001	0.79 (0.70-0.88)	<.001
≥50	0.59 (0.42-0.85)	.00	0.59 (0.51-0.69)	<.001
Unknown	1.61 (0.01-248.33)	.85	0.55 (0.10-2.86)	.48

Abbreviations: NA, not applicable; NCCN, National Comprehensive Cancer Network; OR, odds ratio.

^a^ Pearson χ^2^ test was used to test significance, Wald test hospital-level clustering was used for calculation of SEs.

^b^ Other includes other and unknown as defined within the National Cancer Database.

For kidney cancer, age and both Medicaid (OR, 1.57; 95% CI, 1.30-1.90) and Medicare (OR, 1.33; 95% CI, 1.18-1.50) were associated with increased odds of nonoperative management. Additionally, Black race (OR, 1.49; 95% CI, 1.32-1.69) and an annual income less than $38 000 (OR, 1.24; 95% CI, 1.07-1.44) were associated with increased odds of nonoperative management. In contrast, few comorbidities (CCI scores of 1 and 2; OR, 0.76; 95% CI, 0.68-0.86) were associated with lower odds of nonoperative management.

**Figure 1.**
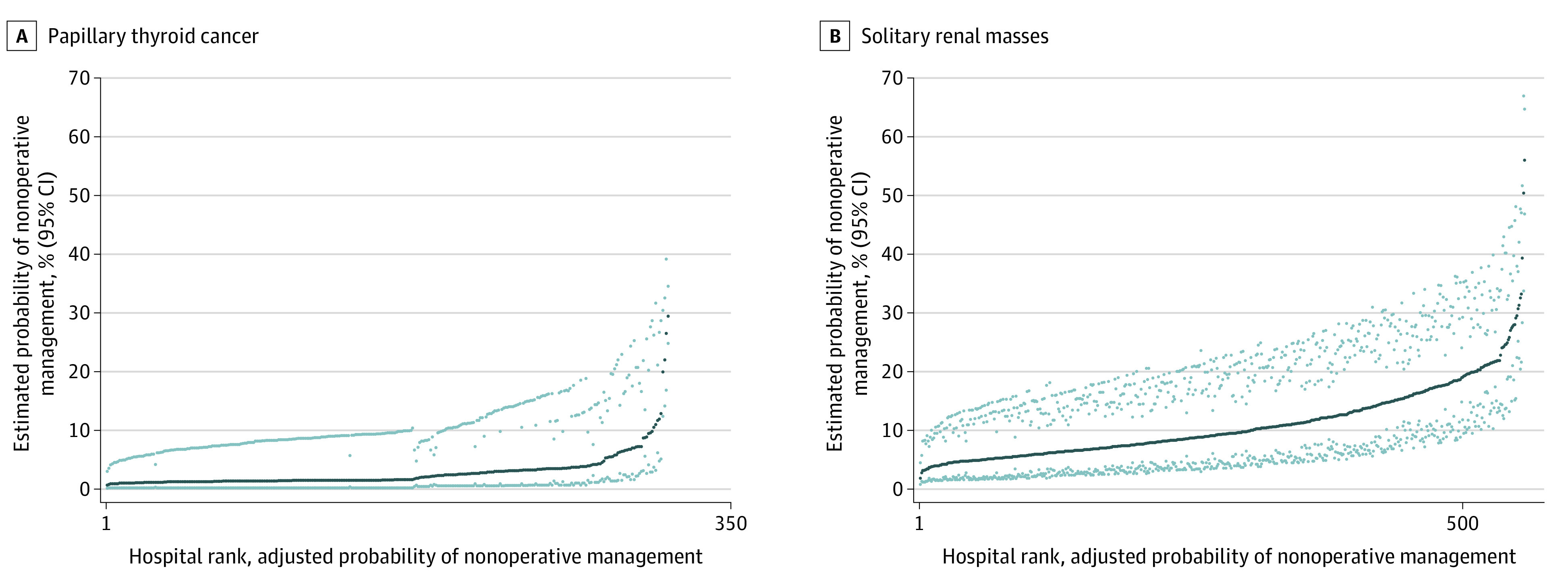
Variability in Risk-Adjusted Hospital-Level Probability of Nonsurgical Management Among Patients With Papillary Thyroid Cancer and Solitary Kidney Masses Eligible for Surveillance Panel A shows papillary thyroid cancer and panel B shows kidney masses. Dark dots represent hospital probability of nonoperative management, and light dots represent 95% CIs.

After adjusting for baseline patient variables, we compared the estimated probability of nonoperative management between hospitals and found significant between-hospital variability. Among patients with thyroid cancer, there were a total of 315 unique hospitals with at least 25 eligible patients for observation, the risk-adjusted probability of nonoperative management ranged from 1.1% in the bottom decile (95% CI, 1.0%-1.1%, range 0.7%-1.2%) to 10.3% in the top decile (95% CI, 8.0%-12.4%, range 5.8%-29.5%). Patients with kidney cancer eligible for nonoperative management were treated at 557 unique hospitals with at least 25 eligible patients for observation. The mean estimated hospital-level probability of nonoperative management ranged from 4.3% in the bottom decile of hospitals (95% CI, 4.1%-4.4%; range, 1.9%-4.9%) to 24.6% in the top decile of hospitals (95% CI, 22.7%-26.5%; range, 19.5%-56.1%). Of the limited hospital variables available—cancer center designation, case volume (mean [SD] number of cases, 125.42 [94.28] for kidney cancer and 108.169, [105.32] for thyroid cancer), and geographic region—none was significantly associated with higher rates of nonoperative management. The facility type, location of the hospital in the US, and geographical area (urban vs rural) were not associated with lower rates of nonoperative management. Ranked caterpillar plots showing the adjusted hospital-level probabilities of nonsurgical management from lowest to highest in the 2 cancers are shown in Figure 1.

**Figure 2.**
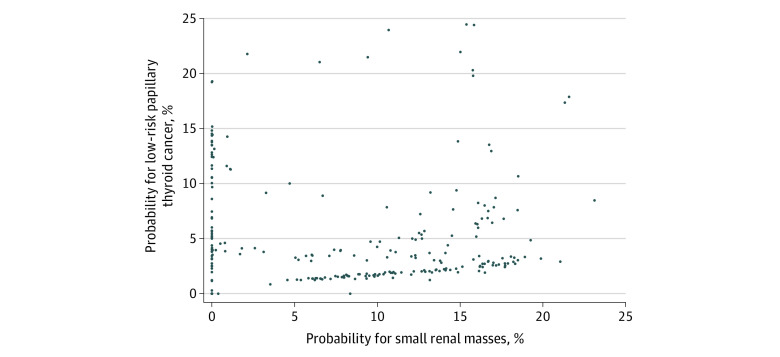
Hospitals’ Probability of Nonsurgical Management for Low-risk Papillary Thyroid Cancer vs Small Kidney Masses

In our combined analysis, we identified 262 hospitals with at least 25 patients eligible for nonoperative management in both cancers. A scatterplot of each of these hospitals’ unadjusted probabilities (observed proportions) of nonoperative management for thyroid vs kidney cancer is shown in Figure 2. The estimated hospital-level probabilities of nonoperative management between the 2 cancers were weakly to minimally correlated and statistically significant (Spearman ρ = 0.33; *P* < .001). In other words, for patients with thyroid or kidney cancer treated at the same hospital, the estimated probability of receiving nonoperative management for eligible patients with thyroid cancer was only weakly correlated with the probability of nonoperative management for patients with kidney cancer. The adjusted mixed-effects model for the risk-adjusted hospital-level probabilities of nonoperative management between the 2 cancers was also correlated and statistically significant (Spearman ρ = 0.32; *P* < .001).

## Discussion

In this retrospective, hospital-based cross-sectional study of individuals with a diagnosis of kidney or thyroid cancer who met stage and grade criteria for nonoperative management, nonoperative management was used in only a minority of cases. In both adjusted and unadjusted analyses, we saw significant between-hospital variability in the probability of nonoperative management. In thyroid cancer, there was a 10-fold difference between the top and bottom decile. In kidney cancer, there was a more than 6-fold difference. In other words, demographically and clinically similar patients with low-risk thyroid and kidney cancer eligible for surveillance have vastly different probabilities of nonoperative management according to where they receive care. Additionally, we found overlap in the patient-level demographic and clinical variables associated with increased probability of nonoperative management. In both cancers, age was associated with increased probability of nonoperative management, whereas female sex and low comorbidity scores were associated with decreased probability of nonoperative management within thyroid cancer. Within kidney cancer, Medicaid and Medicare were associated with increased odds of nonoperative management. Black race and an income less than $38 000 were associated with increased odds of nonoperative management. Regarding whether the probabilities of observation in the 2 cancers are correlated, we found that hospitals’ probability of nonoperative management of papillary thyroid cancer had a weak to minimal but statistically significant correlation with their probability of nonoperative management of small kidney masses.

### Limitations and Strengths

How often endocrine and urologic surgeons in the same institution use nonoperative management is certainly multifactorial. Considering that nonoperative approaches are new within thyroid cancer treatment, some factors are likely to be similar across different departments within the same institutions and within the same referral regions, whereas others may be specialty and disease specific. Culture, practice patterns, and established treatment regimens may encourage physicians working in the same department to treat cancer more aggressively. In contrast, financial reimbursement of the physicians (often similar at a given hospital) may be associated with the odds of receiving certain treatments equally.^22,23,24^ Regarding the overall low implementation of observational treatment within both cancers, capital investment in expensive technology and surgical infrastructure can lead to economic pressure to operate more.^25^ Market competition may be another factor. Wright et al^26^ have shown that regional market forces play a large role in determining which hospitals see the greatest diffusion of certain surgical techniques, such as robotic surgery. An extension of this phenomenon is the potential for surgeons to operate more (or less) to keep up with local competitors and be at the forefront of medical care. Additionally, highly health-literate patients may travel to high-volume institutions specifically looking for expert surgeons, and some large hospital centers may market their surgical expertise throughout regional and national markets, which would account for this. Conversely, highly health-literate patients and financially flexible patients may seek out active surveillance programs in thyroid cancer and urologic oncology.^27^ Given the nature of these data, this study cannot identify the specific reasons for choosing nonoperative management vs surgery in each case. Data on physician and patient beliefs, as well as more granular aspects of patients’ social setting which may be relevant to decisions about treatment vs surveillance, are all potential confounders and are not available.

In addition to lack of granular data and unmeasured confounders there are other important limitations to this study. For one, the National Cancer Database is not a truly population-based data set. Although the database does capture more than 92.1% and 86.7% of all the thyroid and kidney cancers, respectively, in the US,^11^ the findings may not be fully generalizable to the larger population. Nonoperative management may sometimes take place in a nonhospital-based setting. We also omitted prostate cancer, because capture rates for prostate cancer in the NCDB are substantially lower than thyroid and kidney (50.8% of all US cases).^11^

Despite these limitations, our study provides strong, methodologically rigorous data describing variability in nonoperative management within and between hospitals, and across seemingly unrelated cancers. Using the random effects term, which encompasses the hospital-level variability due to measured and unmeasured hospital characteristics, we can define the contribution of hospitals that is above and beyond the individual patients’ characteristics. This analysis also provides estimates for probability of nonoperative management based entirely on the contribution of each hospitals’ characteristics, while holding patient level characteristics constant.

## Conclusions

In this cross-sectional study of individuals with a diagnosis of kidney or thyroid cancer who met stage and grade criteria for nonoperative management, our results suggest that there remained significant between-hospital variability in use of nonoperative management for low-risk cancers that was not fully explained by clinical and pathological factors. Furthermore, the weak to minimal correlation of nonoperative management in thyroid and kidney cancer within hospitals suggests that hospital factors which cut across multiple specialties and departments are likely to play some role in odds of surgical management within institutions, but this is likely not the whole story; patient, surgeon, and departmental factors all play a role in the complex and multifaceted decision about whether to offer nonoperative management in these cases.

## Notes

Supplement. **eTable 1.** Stage and Grade Criteria of Patients Eligible for Observation and Criteria for Assigning Observation **eTable 2.** Patient-Level Predictors of Nonoperative Management Among Eligible Patients With Low-Risk Papillary Thyroid Cancer and Small Kidney Masses Using Generalized Estimating Equations With a Compound Symmetry Correlation Structure
