# Supplementary material for: Understanding Hospital-Level Patterns of Nonoperative Management for Low-risk Thyroid and Kidney Cancer
Source: JAMA Netw Open. 2022 Nov 15;5(11):e2242210. doi: 10.1001/jamanetworkopen.2022.42210 (PMC9667332; doi:10.1001/jamanetworkopen.2022.42210)
Supplement: Supplement. — eTable 1. Stage and Grade Criteria of Patients Eligible for Observation and Criteria for Assigning Observation eTable 2. Patient-Level Predictors of Nonoperative Management Among Eligible Patients With Low-Risk Papillary Thyroid Cancer and Small Kidney Masses Using Generalized Estimating Equations With a Compound Symmetry Correlation Structure [file jamanetwopen-e2242210-s001.pdf]

Supplemental Online Content

Koelker M, Krimphove M, Alkhatib K, et al. Understanding hospital-level patterns of nonoperative management for low-risk thyroid and kidney cancer. *JAMA Netw Open*. 2022;5(11):e2242210. doi:10.1001/jamanetworkopen.2022.42210

eTable 1. Stage and Grade Criteria of Patients Eligible for Observation and Criteria for Assigning Observation
eTable 2. Patient-Level Predictors of Nonoperative Management Among Eligible Patients With Low-Risk Papillary Thyroid Cancer and Small Kidney Masses Using Generalized Estimating Equations With a Compound Symmetry Correlation Structure

This supplemental material has been provided by the authors to give readers additional information about their work.

© 2022 Koelker M et al. *JAMA Network Open.*

eTable 1: Stage and Grade Criteria of Patients Eligible for Observation and Criteria for Assigning Observation

	Histology	Stage/Size, Grade Criteria	Criteria for Surveillance/Observation
**Thyroid**			
	Papillary	cT1a (= <1cm)<br><br>Excluding:<br>- clinical metastasis M+<br>- clinical N+	No surgery and active surveillance
**Kidney**			
	N/A	cT1a (= <4cm)<br><br>Excluding:<br>- clinical metastasis M+<br>-clinical N+	No surgery and active surveillance

© 2022 Koelker M et al. *JAMA Network Open.*

eTable 2: Patient-Level Predictors of Nonoperative Management Among Eligible Patients With Low-Risk Papillary Thyroid Cancer and Small Kidney Masses Using Generalized Estimating Equations With a Compound Symmetry Correlation Structure

	**Papillary Thyroid Cancer**		**Solitary Renal Masses**	
	Coefficient (95%CI)	P value*	Odds Ratio (95%CI)	P value*
**Sex**				
Male	*Ref*		*Ref*	
Female	-0.26 (-0.51- -0.02)	0.04	-0.04 (-0.12-0.05)	0.35
**Age Group**				
≤ 49	*Ref*		*Ref*	
50-59	0.03 (-0.26-0.33)	0.82	0.40 (0.21-0.60)	<0.001
60-69	0.28 (-0.04-0.61)	0.09	0.81 (0.62-0.99)	<0.001
70-79	0.43 (-0.03-0.89)	0.07	1.41 (1.21-1.62)	<0.001
80-89	1.81 (1.28-2.35)	<0.001	2.58 (2.35-2.80)	<0.001
**Race**				
White	*Ref*		*Ref*	
Aanphi	0.21 (-0.21-0.62)	0.33	-0.31 (-0.62-0.01)	0.06
Black	-0.10 (-0.54-0.35)	0.66	0.41 (0.29-0.53)	<0.001
Other	0.40 (-0.08-0.87)	0.10	-0.10 (-0.40-0.20)	0.51
**Charlson Comorbidity Index**				
0	*Ref*		*Ref*	
1	-0.53 (-0.93- -0.14)	0.01	-0.28 (-0.40- -0-17)	<0.001
≥2	-0.39 (-0.98-0.21)	0.20	-0.18 (-0.30- -0.06)	0.00
**Insurance**				
Private	*Ref*		*Ref*	
Medicare	0.02 (-0.33-0.37)	0.92	0.31 (0.19-0.43)	<0.001
Medicaid	0.04 (-0.38-0.45)	0.87	0.54 (0.35-0.74)	<0.001
Other	0.27 (-0.22-0.77)	0.28	0.35 (0.13-0.56)	0.00
**Family Income**				
> $63,000	*Ref*		*Ref*	
$48,000-62,999	-0.30 (-0.60-0.01)	0.06	0.11 (-0.01-0.24)	0.08
$38,000-47,999	-0.09 (-0.42-0.23)	0.57	0.16 (0.03-0.30)	0.02
<$38,000	-0.59 (-1.03- -0.15)	0.01	0.32 (0.17-0.46)	0.01
unknown	-0.87 (-5.06-3.33)	0.68	0.63 (-0.92-2.19)	0.42
**Distance to Hospital (miles)**				
0-12.4	*Ref*		*Ref*	
12.5-49.9	-0.95 (-1.25- -0.65)	<0.001	-0.23 (-0.33- -0.12)	<0.001
≥ 50	-0.50 (-0.89-0.10)	0.01	-0.55 (-0.70- -0.40)	<0.001
unknown	-0.00 (-4.21-4.21)	1.00	-0.70 (-2.25-0.86)	0.38

*Pearson’s chi-squared test was used to test significance, Wald test hospital level clustering was employed for calculation of standard errors

© 2022 Koelker M et al. *JAMA Network Open.*
